# BMAL1 sex‐specific effects in the neonatal mouse airway exposed to moderate hyperoxia

**DOI:** 10.14814/phy2.16122

**Published:** 2024-06-28

**Authors:** Colleen M. Bartman, Lisa Nesbitt, Kenge K. Lee, Latifa Khalfaoui, Yun‐Hua Fang, Christina M. Pabelick, Y. S. Prakash

**Affiliations:** ^1^ Department of Anesthesiology and Perioperative Medicine Mayo Clinic Rochester Minnesota USA; ^2^ Department of Physiology & Biomedical Engineering Mayo Clinic Rochester Minnesota USA

**Keywords:** airway hyperresponsiveness, asthma, BMAL1, clock biology, hyperoxia

## Abstract

Supplemental O_2_ (hyperoxia) is a critical intervention for premature infants (<34 weeks) but consequently is associated with development of bronchial airway hyperreactivity (AHR) and asthma. Clinical practice shifted toward the use of moderate hyperoxia (<60% O_2_), but risk for subsequent airway disease remains. In mouse models of moderate hyperoxia, neonatal mice have increased AHR with effects on airway smooth muscle (ASM), a cell type involved in airway tone, bronchodilation, and remodeling. Understanding mechanisms by which moderate O_2_ during the perinatal period initiates sustained airway changes is critical to drive therapeutic advancements toward treating airway diseases. We propose that cellular clock factor BMAL1 is functionally important in developing mouse airways. In adult mice, cellular clocks target pathways highly relevant to asthma pathophysiology and *Bmal1* deletion increases inflammatory response, worsens lung function, and impacts survival outcomes. Our understanding of BMAL1 in the developing lung is limited, but our previous findings show functional relevance of clocks in human fetal ASM exposed to O_2_. Here, we characterize *Bmal1* in our established mouse neonatal hyperoxia model. Our data show that *Bmal1* KO deleteriously impacts the developing lung in the context of O_2_ and these data highlight the importance of neonatal sex in understanding airway disease.

## 1 INTRODUCTION

The sensitivity of developing airways to oxygen is well documented both clinically and through numerous laboratory studies. During gestation, the fetus experiences an oxygen tension that creates a relatively hypoxic environment compared to the air we breathe (21% O_2_ or 160 mmHg): fetal PaO_2_ (partial pressure of oxygen in the arterial blood) is ~25–50 mmHg and maternal PaO_2_ is ~80–90 mmHg (Simon & Keith, 2008; Torres‐Cuevas et al., 2017). As fetal development progresses, oxygen tension increases, although still within a relatively hypoxic range (i.e., embryonic development requires lower PaO_2_ compared to fetal development) (Simon & Keith, 2008). Transitioning from *in utero* to the *ex utero* environment requires the lungs be adequately equipped to handle this sudden and dramatic increase in oxygen availability, which presents a relatively hyperoxic environment (Dawson et al., 2010). Thus, preterm birth (<34 weeks gestation) poses a significant challenge for underdeveloped lungs ill‐equipped to handle high levels of oxygen. Additionally, supplemental O_2_ is often administered to premature infants in the neonatal ICU (NICU), further exacerbating the hyperoxic insult on the lungs (Bhandari, 2010; Dawson et al., 2010; Jobe & Kallapur, 2010; Velten et al., 2010). Clinical practice has shifted away from the use of high O_2_ concentrations (80%–90%) in the NICU since it is now known to promote bronchopulmonary dysplasia (BPD) (Saugstad & Aune, 2011; Vento et al., 2009), but even moderate O_2_ (<60%) still poses a problem for the developing lung.

Current practice implements moderate oxygen supplementation (30%–60% O_2_) (Finer & Leone, 2009; Saugstad & Aune, 2011), but even moderate hyperoxia promotes subsequent airway disease with chronic inflammation, AHR, and remodeling (Baldwin & Roche, 2002; Been et al., 2014; Di Fiore et al., 2019; Dylag et al., 2017; Martin et al., 2013; Saglani et al., 2007; Vogel et al., 2015; Wang et al., 2014). Thus, early hyperoxic insults combined with preterm birth drive airway narrowing, increased AHR, higher likelihood of obstruction due to structural changes (Haland et al., 2006; Hovland et al., 2013; Pike et al., 2011), and ultimately pediatric asthma (Baldwin & Roche, 2002; Barbato et al., 2003; Malmstrom et al., 2013; Saglani et al., 2007). Studies show that altered airway structure and function in neonates contributes to childhood asthma: children less than 3 years old with thickened airways display recurrent wheeze prior to the emergence of pediatric asthma (Baldwin & Roche, 2002; O'Reilly et al., 2013; Saglani et al., 2007). Additionally, in vitro studies using human fetal ASM exposed to moderate O_2_ show increased intracellular calcium, proliferation, extracellular matrix deposition, and cellular senescence (Britt Jr. et al., 2015; Hartman et al., 2012; Parikh et al., 2018; Roesler et al., 2021). Similarly, in vivo studies using a neonatal mouse model of moderate hyperoxia exposure show increased ASM thickness, collagen deposition, AHR following methacholine (MCh) challenge, and sex differences in response to O_2_ (Bartman et al., 2024; Faksh et al., 2016; Wang et al., 2014). Because hyperoxic insults with prematurity have immediate and long‐term impacts for the lung, understanding the mechanisms of moderate O_2_ effects could be pivotal for identifying targetable mechanisms for improved therapeutic strategies.

We propose that BMAL1, a core component of peripheral cellular clocks (i.e., circadian rhythms) links early oxygen exposure to airway structure and function. BMAL1 (brain and muscle ARNT‐like 1; formerly ARNTL, aryl hydrocarbon receptor nuclear translocator‐like protein 1) is a basic‐helix–loop–helix‐PAS (Per‐Arnt‐Sim) domain protein that is well characterized for its role in circadian rhythmicity in adults (Allada et al., 2001; Bartman et al., 2020; Bunger et al., 2000; Hogenesch et al., 1998). BMAL1 forms a heterodimer with CLOCK and together functions as a transcription factor complex that interacts with genomic enhancer regions to regulate expression of downstream “clock‐controlled genes” (Allada et al., 2001; Bunger et al., 2005; Hogenesch et al., 1998). These clock‐controlled genes encode products that either are involved in the cellular clock feedback loop (generating rhythmic gene expression of other clock components *Per1/2*, *Cry1/2*) or are direct regulators of basic cellular and physiological processes that maintain homeostasis and overall function (Albrecht, 2004; Dibner et al., 2010). Importantly, these pathways are highly relevant to developing airways and lung function (Bartman et al., 2020). Peripheral cellular clocks are expressed in all cell types of the lung (Albrecht, 2004; Dibner et al., 2010), and clock networks are implicated in the pathophysiology of adult asthma (Bartman et al., 2020; Nosal et al., 2020). Previous studies clearly document a role for BMAL1 in adult lung function: adult *Bmal1* KO mice infected with influenza A have increased inflammation, reduced viral clearance, and poorer survival outcomes (Sengupta et al., 2019), and adult *Bmal1* KO mice show exaggerated asthma‐like changes in the airway, such as increased mucus production and increased airway resistance to MCh challenge, compared to mice with both *Bmal1* alleles (Ehlers et al., 2018). Influenza A infection of mice with *Bmal1* deleted in club cell secretory protein expressing pulmonary airway epithelial cells increases bronchoalveolar neutrophils, reduces lung function, and results in poorer response to infection compared to *Bmal1* WT mice receiving the infection (Zhang et al., 2019). There is recent (albeit limited) exploration of clocks in adult airway diseases, but the role of clock biology in developing airways is largely unknown.

There are three studies relevant to BMAL1 and perinatal development. Using a BPD model of neonatal mice exposed to high O_2_ (95%), researchers demonstrated long‐term effects of O_2_ on lung function following infection administered in adulthood: mice that received high O_2_ as neonates had altered clock networks and inflammatory response to adulthood infection (Issah et al., 2021). Furthermore, deletion of *Bmal1* in alveolar Type 2 cells had a similar effect on lung function in response to adult infection compared to adult mice exposed to hyperoxia as neonates (Issah et al., 2021). Another elegant study demonstrated the importance of BMAL1 during fetal development. This group generated a conditional *Bmal1* KO mouse model where *Bmal1* remained present during fetal development and was deleted postnatally. While the classic *Bmal1* KO mouse model has a distinct phenotype (lacks circadian rhythmicity in behavior [wheel‐running activity, sleeping; eating], reduced lifespan, infertility, sarcopenia, arthropathy, joint ankylosis, and increased inflammation (Bunger et al., 2000; Bunger et al., 2005; Kondratov et al., 2006; Yang et al., 2016)), postnatal deletion of *Bmal1* (i.e., fetal expression maintained) restored many of these aging‐related pathologies (Yang et al., 2016). The importance and complexity of cellular clocks is particularly apparent in work such as this involving prenatal development. Lastly, previous work from our group used human fetal ASM to investigate the role of cellular clock biology in mechanisms of moderate O_2_ exposure. We showed that human fetal ASM express core clock genes/proteins and can be synchronized in culture, similar to responsiveness in human adult ASM (Bartman et al., 2021). Furthermore, knockdown of core clock factors, including *Bmal1*, altered intracellular calcium regulation in moderate hyperoxia which could be rescued by targeting clock proteins (Bartman et al., 2021). These studies highlight the importance of BMAL1 in lung function, the long‐term effects of oxygen exposure during the neonatal period, and the potential role for BMAL1 in chronic airway diseases in neonates and children.

In the present study, we hypothesize that clock component BMAL1 serves a functional role in the developing mouse lung and that moderate hyperoxia would exacerbate these effects. We use the global *Bmal1* WT, Het, and KO mouse model and introduce these neonates, both males and females, to our established moderate hyperoxia exposure model (Bartman et al., 2024; Faksh et al., 2016; Vogel et al., 2019; Wang et al., 2014). Here, we focus on *Bmal1* in developing airways by characterizing airway structure and function in the morning from *Bmal1* WT, Het, and KO male and female neonates with or without moderate O_2_ exposure. These studies suggest that *Bmal1* KO contributes to a greater adverse effect on neonatal hyperoxia‐mediated lung injury for male mice, but there is lower susceptibility to injury in female *Bmal1* KO mice. These data show a role of *Bmal1* in neonatal lung mechanics and airway structure with differences based on sex and O_2_ exposure.

## 2 MATERIALS AND METHODS

### 2.1 Animal model

All animal studies were approved by the Institutional Animal Care and Use Committee at Mayo Clinic and performed in accordance with the National Institutes of Health's Guide for the Care and Use of Laboratory Animals. *Bmal1* ^+/+^ (homozygous WT), *Bmal1* ^+/−^ (heterozygous; WT/KO genotype), and *Bmal1* ^−/−^ (homozygous KO) mice were obtained as a generous gift from Dr. Aleksey Matveyenko at Mayo Clinic, the strain of which originated from The Jackson Laboratory (B6.129‐*Arntl* ^tm1Bra^/J; JAX stock #009100) (Bunger et al., 2000, 2005). Adult *Bmal1* WT and *Bmal1* Het mice were crossed to expand the mouse colony. It is known that BMAL1 has a critical role in fertility since adult *Bmal1* KO mice are infertile (Alvarez et al., 2008; Boden et al., 2010; Liu et al., 2014; Schoeller et al., 2016; Tonsfeldt et al., 2019; Yang et al., 2016). For experiments, adult *Bmal1* Het mice were crossed to generate approximately 50% Het, 25% WT, and 25% *Bmal1* KO pups. Our goal was to investigate the independent role of BMAL1 as a factor in neonatal hyperoxic lung injury, not its involvement in circadian rhythmicity. However, because BMAL1 is inherently involved in the circadian system, we restricted our studies to one time range: the morning (i.e., all lung mechanics tests and tissue harvesting were done between 8 am and 12 pm). Additionally, *Bmal1* WT, Het, and KO mouse pups are in general visually indistinguishable between genotypes as pups. The *Bmal1* KO genotype becomes very clearly apparent as the mice age, due to the *Bmal1* KO “early‐aging” phenotype and multitude of aging‐related pathologies (Kondratov et al., 2006; Yang et al., 2016). Because we expose neonates to the hyperoxia chamber on P0 and genotyping cannot occur until ~P21, we cannot control how many pups of each genotype and sex were divided among 21% or 50% O_2_, resulting in a skewed distribution of animals per genotype/sex/O_2_ exposure group. Through the process of generating sufficient KOs of both males and females, we generate an abundance of Het neonates. Once each group had at least the minimum number of animals (*n* = 3), we stopped breeding. Even though this resulted in an uneven distribution of genotypes in each condition, we felt it would be wasteful and unethical to attempt generating additional KOs at the expense of an abundance of Hets. *Bmal1* genotyping was done using TransnetYX (Memphis, Tennessee, USA) following tail clipping before P21 and marking neonates with a felt tipped marker. Male and female neonates were used in the studies.

### 2.2 Hyperoxia exposure

Neonatal hyperoxia was performed as previously described (Bartman et al., 2024; Vogel et al., 2019; Wang et al., 2014). Constant temperature and light/dark cycles (12:12 light: dark) were maintained. Food (irradiated PicoLab Rodent Diet 20; LabDiet 5053) and water were provided ad libitum. Newborn pups (P0) were exposed to either room air at 21% O_2_ (normocapnic, isobaric normoxia) or 40%–50% O_2_ (normocapnic and isobaric hyperoxia). Hyperoxia exposure was done using a custom built, sealed plexiglass chamber with an inlet and a pressure relief valve, and hyperoxic O_2_ was delivered via an oxygen/air blender. Appropriate O_2_ levels were monitored using a digital oxygen monitor. Dams were alternated between 21% and 50% O_2_ on a daily basis to prevent maternal O_2_ toxicity. Neonates in the hyperoxia group were exposed to 50% O_2_ from P0 to P7, after which there was a 14‐day recovery period in 21% O_2_. At P21, neonates were subject to pulmonary function testing (flexiVent) under anesthesia (ketamine/xylazine). Mice were euthanized by anesthetic overdose and exsanguination, and tissue was harvested for structural studies (histology) and protein expression studies (Jess).

### 2.3 Lung mechanics

*Bmal1* WT, Het, and KO neonates were assessed for lung mechanics using flexiVent (Scireq; Montreal, Canada) after the neonatal hyperoxia exposure model at P21. Airway resistance (Rrs), compliance (Crs), and elastance (Ers), and tissue damping (G) and elasticity (H) were determined as previously described (Bartman et al., 2024; Faksh et al., 2016; Wang et al., 2014). Briefly, experiments were done under anesthesia (ketamine/xylazine) of P21 neonates, and appropriate anesthetic depth verified before administering the paralytic (vecuronium). Body temperature was maintained at 37°C (heating pad underneath the mouse). Tracheostomy was performed and a blunt tip metal cannula inserted and secured using a suture loop around the exposed trachea. Mice were then attached to the flexiVent system for lung mechanics measurements under static and dynamic conditions per manufacturer's protocols. Mice were administered a methacholine (MCh) challenge starting at baseline (0 mg/mL MCh) and followed by increasing doses of nebulized MCh (6.25, 12.5, 25.0, and 50.0 mg/mL). Lastly, while mice remained under anesthesia, lungs were harvested (fixed or frozen) for subsequent analyses.

### 2.4 Histology

Histological analyses were performed as previously detailed (Bartman et al., 2024; Parikh et al., 2018; Vogel et al., 2019). Lungs were harvested (inflated at 25 cmH_2_O with 4% paraformaldehyde) from P21 *Bmal1* WT, Het, and KO neonates from both 21% and 50% O_2_ groups immediately following MCh challenge on the flexiVent. Formalin‐fixed paraffin‐embedded (FFPE) sections were cut at 5 mm for standard hematoxylin (hematoxylin solution, Gill No. 3, Sigma‐Aldrich, GHS332; St. Louis, MO, USA) and eosin (eosin Y, Polysciences, 09859; Warrington, PA, USA) (H&E) stain or Masson's trichrome (Masson's Trichrome Stain Kit, Polysciences, 25,088, Warrington, PA, USA) (MT) staining. Imaging was done using a Motic Easy Scan Digital Slide Scanner (Motic; Hong Kong, China). For H&E, ImageJ (NIH; Bethesda, Maryland, USA) software was used to calculate airway thickness (by measuring airway area and perimeter) from a minimum of three airways per section and a minimum two sections per animal (Bartman et al., 2024; Parikh et al., 2018; Vogel et al., 2019). Airways were quantified regardless of shape or size to minimize bias. For MT, Orbit Image Analysis Software (Idorsia Pharmaceuticals Ltd.; Allschwil, Switzerland) was used to measure percent collagen within airways by implementing a pixel‐based classification training model that differentiates the amount of collagen (inclusion) versus background (exclusion) (Fitzgerald et al., 2019; Stritt et al., 2020). Regions of interest (airways) were then quantified, and the resulting ratio multiplied by 100. Representative images were white‐balanced with consistent parameters using ImageJ.

### 2.5 Protein expression

Frozen whole lung mouse tissue was homogenized in sucrose buffer (250 mM sucrose, 40 mM Tris, pH 7.2) supplemented with 1X Protease and Phosphatase Inhibitor Cocktail (ThermoScientific #1861280; Waltham, MA, USA) and using a Bullet Blender Homogenizer at 4°C. Lysate was centrifuged at 12,000 rpm for 10 min at 4°C. Protein from supernatant was quantified using a DC Protein Assay kit (Bio‐Rad, 5000111; Hercules, CA, USA) and FlexStation plate reader. Protein expression was measured using Jess Automated Western Blot System by Protein Simple (ProteinSimple, Bio‐Techne Brand, #004–650; San Jose, CA, USA), an automated and quantitative digital Western blot technology that uses a capillary immunoassay system for protein quantification. Amount of protein loaded per capillary was either ~0.3 mg (BMAL1, Collagen I, Collagen III, Fibronectin) or ~0.6 mg (PER1), based on antibody optimization. Protein expression was normalized to total protein, which was measured using Total Protein Detection Module for Chemiluminescence (ProteinSimple, Bio‐Techne Brand #DM‐TP01; San Jose, CA, USA), following manufacturer's protocols. The following primary antibodies were used: BMAL1 (D2L7G) Rabbit mAb #14020 (Cell Signaling Technology; Danvers, MA, USA) at 1:25; PER1 Rabbit NBP2‐24589 (Novus Biologicals; Centennial, CO, USA) at 1:50; Fibronectin Rabbit ab2413 (Abcam; Cambridge, UK) at 1:200; Collagen I Rabbit ab34710 (Abcam; Cambridge, UK) at 1:20; Collagen III Rabbit ab7778 (Abcam; Cambridge, UK) at 1:20. BMAL1 peaks were measured at ~86 kDa, PER1 peaks ~50 kDa, collagen I peaks ~150 kDa, collagen III peaks ~280 kDa, and fibronectin peaks ~280 kDa. The ~50 kDa PER1 fragment has been identified before in several other tissues and cell types as an active nuclear fragment that is smaller than full‐length PER1 (Chilov et al., 2001; Gumz et al., 2010; Richards et al., 2012, 2013, 2014; Solocinski et al., 2015). The Anti‐Rabbit Secondary HRP Antibody was used (ProteinSimple, Bio‐Techne Brand, #042‐206; San Jose, CA, USA), which is suppled at a predetermined concentration suitable for Jess Technology and as part of the Anti‐Rabbit Detection Module (ProteinSimple, Bio‐Techne Brand, #DM‐001; San Jose, CA, USA).

### 2.6 Statistical analysis

Data were analyzed using GraphPad Prism 10.0 software (GraphPad Software, San Diego, CA, USA www.graphpad.com). An ordinary two‐way *ANOVA*, with Tukey's multiple comparison test where appropriate, was used. Outliers were determined by Grubb's outlier test. “*n*” values represent number of animals. Data are represented as mean ± SD and *p* < 0.05 used for statistical significance.

## 3 RESULTS

### 3.1 Introducing *Bmal1* WT, Het, and KO mice to the neonatal hyperoxia exposure model

**FIGURE 1:**
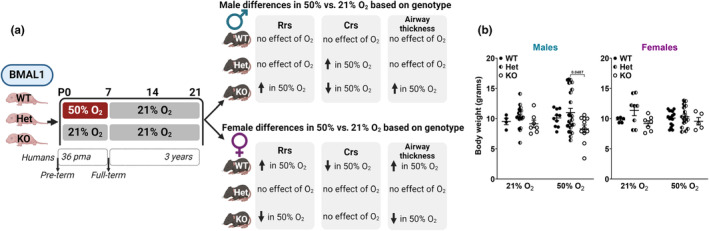
Introducing *Bmal1* WT, Het, and KO mice to the neonatal hyperoxia exposure model. (a) *Bmal1* WT, Het, and KO neonates were exposed to either 21% or 50% O_2_ from P0 to P7 followed by all neonates exposed to 21% O_2_ from P7 to P21 (“recovery” period). P21 pups underwent lung mechanics testing with MCh challenge (flexiVent) and lung tissue harvested for histological (H&E and MT) and molecular (protein expression) analyses. Image created with a licensed version of BioRender.com. BMAL1, brain and muscle arnt‐like; Het, heterozygous; KO, knockout; pma, post menstrual age; WT, wildtype. (b) Neonatal mice were weighed on P21 prior to experimental studies. Ordinary two‐way *ANOVA* was used to compare body weights based on genotype and O_2_ exposure group for each sex. Data are represented as mean ± SD; *n* = 4–24 pups per group.

We previously established a model of moderate hyperoxia exposure in neonatal mice (Bartman et al., 2024; Faksh et al., 2016; Roesler et al., 2021; Vogel et al., 2019; Wang et al., 2014). Briefly, newborn mouse pups (postnatal day 0, P0) are exposed to either 21% O_2_ (room air) or 50% O_2_ (moderate hyperoxia chamber) from P0‐P7. Then, both hyperoxia and normoxia pups are kept at room air from P8‐P21 during the “recovery” period, essentially modeling premature moderate hyperoxia exposure and recovery prior to lung function analysis at P21 (representing ~3 year old child exhibiting wheezing disorders such as asthma) (Figure 1a).

Of 131 neonates resulting from breeding *Bmal1* Hets, our yield was 49.6% Het (24 females and 41 males), 26.7% WT (21 females and 14 males), and 23.7% KO (12 females and 19 males) from our experimental studies. We noticed a distinct phenotype in ~16% of the *Bmal1* KO mouse pups: there were five *Bmal1* KO pups with noticeably smaller stature that was quite apparent even compared to other *Bmal1* KO littermates. Three of these KO pups were males exposed to 50% O_2_ and two of these KO pups were females exposed to 21% O_2_. Although every pup that displayed this phenotype was a *Bmal1* KO (i.e., this phenotype was not evident in any of either the Bmal1 Het or WT neonates), not every *Bmal1* KO had these characteristics. These noticeably smaller pups struggled to gain sufficient weight by P21 or had slowed weight gain compared to its Het or WT littermates (data not shown). Some of the KO pups failed to gain enough weight by P21 that is needed for lung mechanics analysis on the flexiVent (>10 g) or these pups died naturally by P21 (in which case lungs were harvested for structural and molecular analyses). Previous studies reported that *Bmal1* KO mouse pups are visually indistinguishable from their littermates but that the distinct characteristics of adult *Bmal1* KO mice only become pronounced with age: starting around 16–18 weeks of age for both male and female mice, *Bmal1* KO body weight growth rate slows and then begins to decline (Kondratov et al., 2006). Thus, adult *Bmal1* KO mice have reduced body weight compared to WT and Het mice (Kondratov et al., 2006). We noticed distinctively smaller *Bmal1* KO pups, males in hyperoxia and females in normoxia, whose overall size presented challenges for lung function testing at P21. However, among all animals regardless of smaller stature, differences in body weight between male and female neonates based on genotype were not evident. Although, *Bmal1* KO male neonates exposed to 50% O_2_ had lower body weight compared to Het males also exposed to 50% O_2_ (Figure 1b).

**FIGURE 2:**
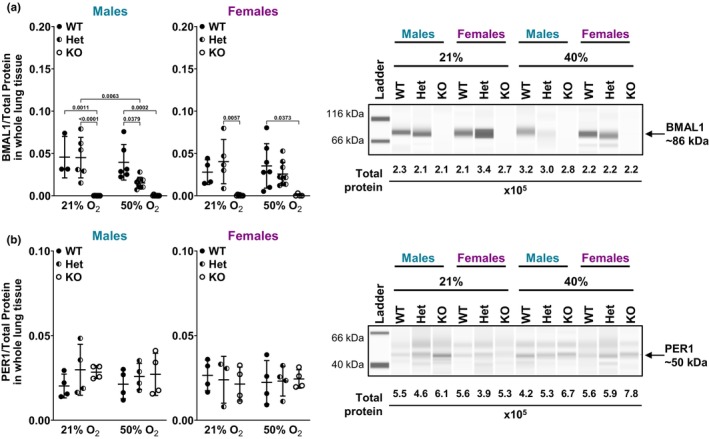
*Bmal1* WT, Het, and KO protein expression validation. (a) BMAL1 protein expression from *Bmal1* WT, Het, and KO male and female neonatal whole lung mouse tissue measured by Jess automated and quantitative immuno‐capillary assay that generates a digital Western blot (representative image shown). ~0.3 mg protein loaded per capillary. BMAL1 peaks were identified using a molecular weight of ~86 kDa. Protein expression was normalized using chemiluminescence of total protein (values indicated on representative image). Ordinary two‐way *ANOVA* was used to compare *Bmal1* genotype and O_2_ exposure groups in each sex. Data are represented as mean ± SD; *n* = 3–9 pups per group. (b) PER1 protein expression using ~0.6 mg protein loaded per capillary based on antibody optimization in Jess. PER1 peaks were identified at a molecular weight of ~50 kDa. Protein expression was normalized using chemiluminescence of total protein (values indicated on representative image). Ordinary two‐way *ANOVA* was used to compare *Bmal1* genotype and O_2_ exposure groups in each sex. Data are represented as mean ± SD; *n* = 3–4 pups per group.

To validate deletion of BMAL1 in *Bmal1* KO mouse lungs and confirm expression of BMAL1 in WT and Het mouse lungs, whole lung tissue was used for protein expression analysis using immuno‐capillary digital Western blot technology. Both WT and Het male and female whole mouse lungs express BMAL1 protein in 21% and 50% O_2_ with BMAL1 undetectable in whole lung tissue from KO mice (Figure 2a). Core clock protein PER1 expression was also evaluated in whole lung tissue from *Bmal1* WT, Het, and KO male and female neonates, at ~50 kDa, without observable significant differences (Figure 2b). Others have shown in several tissues and cell types that PER1 exists as an active ~50 kDa nuclear fragment, which is shorter than full‐length PER1 (Chilov et al., 2001; Gumz et al., 2010; Richards et al., 2012, 2013, 2014; Solocinski et al., 2015).

### 3.2 Effect of *Bmal1*, moderate O_2_ , and neonatal sex on airway resistance (Rrs)

**FIGURE 3:**
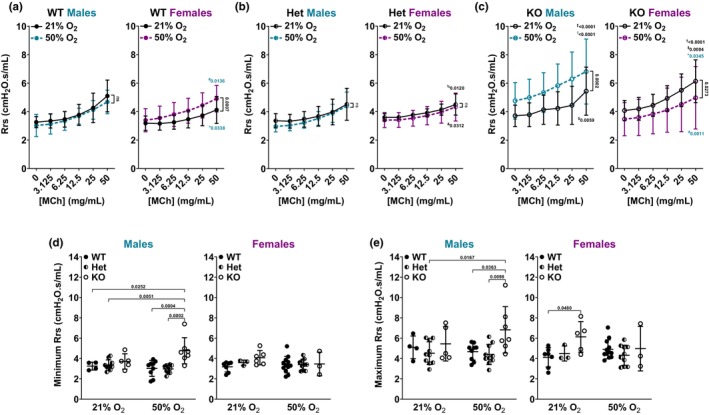
Airway resistance. Lung mechanics (flexiVent) analysis of P21 male and female neonates during MCh challenge of (a) *Bmal1* WT neonates, (b) *Bmal1* Het neonates, and (c) *Bmal1* KO neonates. Effect of 21% versus 50% O_2_ in each sex is shown pairwise on graphs. **+**, Effect of female versus male in 21% O_2_ (same genotype in each comparison; represented in blue on female graph); **#**, Effect of female versus male in 50% O_2_ (same genotype in each comparison; represented in blue on female graph). **%**, Effect of Het versus WT in 21% O_2_ (same sex in each comparison); **&**, Effect of Het versus WT in 50% O_2_ (same sex in each comparison). **†**, Effect of KO versus WT in 21% O_2_ (same sex in each comparison); **‡**, Effect of KO versus WT in 50% O_2_ (same sex in each comparison). **$**, Effect of KO vs Het in 21% O_2_ (same sex in each comparison); **!**, Effect of KO versus Het in 50% O_2_ (same sex in each comparison). (d) Minimum (0 mg/mL MCh) and (e) maximum (50 mg/mL MCh) Rrs values across all groups. (a–c) Two‐way *ANOVA* was used to compare MCh dose response curve of each *Bmal1* genotype and O_2_ exposure group based on sex. (d, e) Two‐way *ANOVA* with Tukey's multiple comparison test was used to compare Rrs at baseline and maximum Rrs response to MCh among *Bmal1* genotypes and O_2_ exposure groups based on sex. (a–e) Data are represented as mean ± SD; *n* = 3–12 pups per group.

**FIGURE 4:**
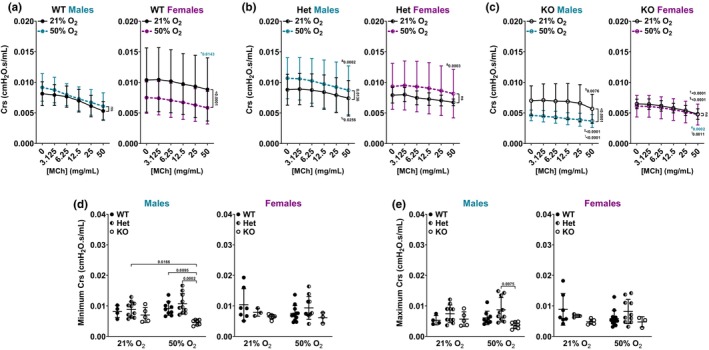
Airway compliance. Lung mechanics (flexiVent) analysis of P21 male and female neonates during MCh challenge of (a) *Bmal1* WT neonates, (b) *Bmal1* Het neonates, and (c) *Bmal1* KO neonates. Effect of 21% versus 50% O_2_ in each sex is shown pairwise on graphs. **+**, Effect of female versus male in 21% O_2_ (same genotype in each comparison; represented in blue on female graph); **#**, Effect of female versus male in 50% O_2_ (same genotype in each comparison; represented in blue on female graph). **%**, Effect of Het versus WT in 21% O_2_ (same sex in each comparison); **&**, Effect of Het versus WT in 50% O_2_ (same sex in each comparison). **†**, Effect of KO versus WT in 21% O_2_ (same sex in each comparison); **‡**, Effect of KO versus WT in 50% O_2_ (same sex in each comparison). **$**, Effect of KO versus Het in 21% O_2_ (same sex in each comparison); **!**, Effect of KO versus Het in 50% O_2_ (same sex in each comparison). (d) Minimum (0 mg/mL MCh) and (e) maximum (50 mg/mL MCh) Crs values across all groups. (a–c) Two‐way *ANOVA* was used to compare MCh dose response curve of each *Bmal1* genotype and O_2_ exposure group based on sex. (d, e) Two‐way *ANOVA* with Tukey's multiple comparison test was used to compare Crs at baseline and maximum Crs response to MCh among *Bmal1* genotypes and O_2_ exposure groups based on sex. (a–e) Data are represented as mean ± SD; *n* = 3–12 pups per group.

Lung mechanics analysis in response to MCh challenge has been done previously in adult *Bmal1* KO mice (Ehlers et al., 2018; Issah et al., 2021; Sengupta et al., 2019; Sundar et al., 2015; Zhang et al., 2019). To our knowledge, a comprehensive lung mechanics assessment of the *Bmal1* WT, Het, and KO neonatal lung following moderate O_2_ exposure has not been done. We previously showed that neonatal mice exposed to moderate O_2_ have increased Rrs, a measurement of the dynamics resistance of the airways (i.e., level of constriction in lungs following MCh challenge) (Faksh et al., 2016; Wang et al., 2014) with differences based on neonatal sex (Bartman et al., 2024). To determine the effect of BMAL1 on developing airways, we characterized lung mechanics (airway resistance (Rrs; Figure 3), compliance (Crs; Figure 4), elastance (Ers; Figure S1a), tissue damping (G; Figure S1b), elasticity (H; Figure S1c), and inspiratory capacity (IC; Figure S1d)) of *Bmal1* WT, Het, and KO neonatal mice following our established moderate hyperoxia exposure model (Figure 1a).

Results showed genotype and sex differences in Rrs following moderate O_2_ exposure and in response to MCh challenge. In considering *Bmal1* WT sex differences in response to MCh challenge, male neonates in 21% O_2_ had higher Rrs compared to females in 21% O_2_, whereas female neonates in 50% O_2_ had higher Rrs compared to males in 50% O_2_ (Figure 3a). WT female neonates exposed to 50% O_2_ from P0 to P7 showed increased Rrs at P21 compared to WT females exposed to 21% O_2_ from P0 to P21, whereas WT males did not demonstrate this effect (Figure 3A). *Bmal1* Het neonates did not demonstrate sex differences in either 21% or 50% O_2_ and neither sex was significantly affected by hyperoxia exposure (Figure 3b). Interestingly, *Bmal1* Het female neonates in 21% O_2_ had higher Rrs compared to WT females in 21% O_2_ while Het females in 50% O_2_ had lower Rrs compared to WT females in 50% O_2_ (Figure 3a,b). *Bmal1* KO neonates showed both sex and O_2_ effects. In males, *Bmal1* KO neonates showed significantly increased Rrs in 50% O_2_ compared to 21% O_2_ (Figure 3c). Furthermore, KO males in 50% O_2_ had higher Rrs compared to WT males in 50% O_2_. KO males in 21% O_2_ and 50% O_2_ showed higher Rrs compared to Het males in both 21% O_2_ and 50% O_2_ (Figure 3b,c). In females, *Bmal1* KO neonates showed decreased Rrs in 50% O_2_ compared to 21% O_2_ (Figure 3c). Furthermore, KO females in 21% O_2_ had higher Rrs compared to WT females in 21% O_2_ (Figure 3a,c). KO females in 21% O_2_ also had higher Rrs compared to Het females in 21% O_2_ (Figure 3b,c). Lastly, there were significant differences between *Bmal1* KO males and females in 21% O_2_ and in 50% O_2_ (Figure 3c).

*Bmal1* KO male neonates exposed to 50% O_2_ had increased baseline (0 mg/mL MCh) Rrs compared to WT and Het males in 21% or 50% O_2_, whereas KO females did not show differences at baseline (Figure 3d). *Bmal1* KO male neonates exposed to 50% O_2_ also had increased maximum (50 mg/mL MCh) Rrs response compared to WT and Het males exposed to 50% O_2_ and compared to Het males in 21% O_2_, whereas KO females in 21% O_2_ had increased maximum Rrs compared to WT females in 21% O_2_ (Figure 3e).

### 3.3 Airway compliance (Crs)

We previously showed that neonatal mice exposed to moderate O_2_ had decreased Crs (Faksh et al., 2016; Wang et al., 2014) with differences based on neonatal sex (Bartman et al., 2024). Crs is inversely proportional to Rrs by measuring the overall elastic property of the respiratory system (i.e., the ease with which it can extend during tidal breathing). In considering *Bmal1* WT sex differences in Crs in response to MCh challenge, male neonates in 21% O_2_ had lower Crs compared to females in 21% O_2_, whereas WT neonates in 50% O_2_ did not show a difference between sexes (Figure 4a). *Bmal1* WT female neonates exposed to 50% O_2_ from P0 to P7 showed decreased Crs at P21 compared to WT females exposed to 21% O_2_ from P0 to P21, whereas WT males did not demonstrate this effect (Figure 4a). *Bmal1* Het neonates did not demonstrate Crs sex differences in either 21% or 50% O_2_. However, Het males had increased Crs in 50% O_2_ compared to Het males in 21% O_2_ (Figure 4b). Additionally, Het males had higher Crs compared to WT males in 21% O_2_ and in 50% O_2_ (Figure 4a,b). Het females in 50% O_2_ had higher Crs compared to WT females in 50% O_2_ (Figure 4a,b). *Bmal1* KO neonates showed both sex and O_2_ effects. In males, *Bmal1* KO neonates showed significantly decreased Crs in 50% O_2_ compared to 21% O_2_ (Figure 4c). KO males in 50% O_2_ had lower Crs compared to WT males in 50% O_2_. KO males in 21% and 50% O_2_ showed lower Crs compared to Het males in 21% O_2_ and 50% O_2_ (Figure 4b,c). In females, *Bmal1* KO neonates did not show a difference in Crs between 21% and 50% O_2_ (Figure 4c). KO females in 21% O_2_ had lower Crs compared to WT females in 21% O_2_ (Figure 4a,c). Additionally, KO females in 21% and 50% O_2_ also had lower Crs compared to Het females in 21% O_2_ and 50% O_2_ (Figure 4b,c). Lastly, *Bmal1* KO males in 50% O_2_ had lower Crs compared to KO females in 50% O_2_ (Figure 4c).

*Bmal1* KO male neonates exposed to 50% O_2_ had decreased baseline (0 mg/mL MCh) Rrs compared to WT and Het males in 50% O_2_ and compared to Het males in 21% O_2_, whereas KO females did not show differences at baseline (Figure 4d). *Bmal1* KO male neonates exposed to 50% O_2_ also had decreased maximum (50 mg/mL MCh) Rrs response compared to Het males in 50% O_2_, whereas KO females did not show significant differences in maximum Rrs among groups (Figure 4e).

Additional parameters obtained from lung mechanics assessment can be found in Figure S1 (https://doi.org/10.6084/m9.figshare.c.7249897): Ers (representing the elastic stiffness of airways following MCh challenge and an indicator of energy conservation in the alveoli to relax to normal size), G (reflects energy dissipation in the tissues and correlates with resistance of the small peripheral airways; higher G values are indicative of greater ASM contraction (Kalidhindi et al., 2019)), H (reflects energy conservation in alveoli), and IC (reflects the amount of air that can be inhaled after the end of normal expiration).

### 3.4 ASM, epithelial, and airway thickness

**FIGURE 5:**
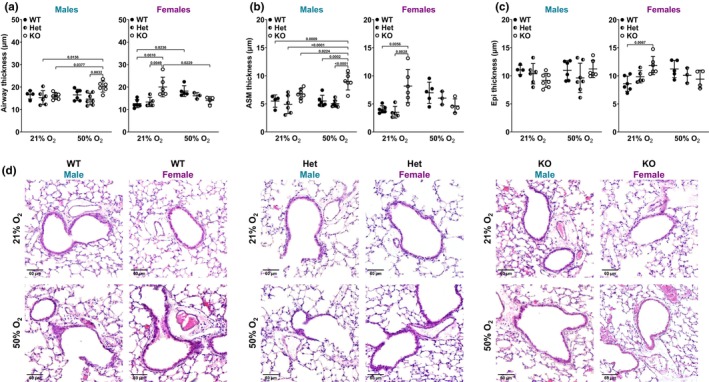
ASM, epithelial, and airway thickness. H&E staining of FFPE sections and quantitative analysis (ImageJ) were used to determine (a) airway thickness, (b) ASM thickness, and (c) epithelial thickness (normalized to airway diameter) of male and female *Bmal1* WT, Het, and KO neonatal mouse lungs. (d) Representative images are shown. ImageJ was used to adjust white balance for consistency. Two‐way *ANOVA* with Tukey's multiple comparison test was used to compare airway, ASM, or epithelial thickness between *Bmal1* genotypes and O_2_ exposure groups based on sex. Data are represented as mean ± SD; *n* = 3–7 per group.

We previously showed that moderate hyperoxia exposure during the neonatal period increases ASM thickness (Bartman et al., 2024; Faksh et al., 2016; Wang et al., 2014). Here, because lung mechanics data revealed differential effects of O_2_ and *Bmal1* genotype based on sex, histological analysis was assessed accordingly. H&E staining showed sex‐ and O_2_‐dependent differences in *Bmal1* genotypes. In *Bmal1* KO male neonates exposed to 21% O_2_, airway thickness was indistinguishable from WT male and Het males also exposed to 21% O_2_ (Figure 5a). 50% O_2_ exposure did not increase airway thickness in WT or Het males. However, KO males exposed to 50% O_2_ from P0 to p7 had increased airway thickness at P21 compared to Het males exposed to 21% or 50% O_2_ and compared to KO males exposed to 21% O_2_ from P0 to P21 (Figure 5a). For females, airways were thicker in *Bmal1* KO neonates in 21% O_2_ compared to WT and Het females in 21% O_2_ (Figure 5a). 50% O_2_ exposure resulted in thicker airways in WT females compared to WT females in 21% O_2_. In contrast to the *Bmal1* KO males, KO female neonates exposed to 50% O_2_ had a decrease in airway thickness compared to KO females in 21% O_2_ and compared to WT females in 50% O_2_, albeit not significant (Figure 5a).

ASM thickness measurements showed a similar pattern. *Bmal1* KO male neonates exposed to 50% O_2_ from P0 to P7 had increased ASM thickness at P21 compared to WT and Het neonates in 21% O_2_ and compared to WT, Het, and KO male neonates in 21% O_2_ (Figure 5b). *Bmal1* WT males exposed to 50% O_2_ did not show an increase in ASM thickness. *Bmal1* KO females showed increased ASM thickness in 21% O_2_ compared to both WT and Het females also in 21% O_2_. *Bmal1* WT females exposed to 50% O_2_ had thicker ASM compared to WT females in 21% O_2_, albeit not significant. Exposing *Bmal1* KO females to 50% O_2_ did not result in increased ASM thickness, which is contrast to *Bmal1* KO males in 50% O_2_ (Figure 5b) and similar to airway thickness measurements (Figure 5a). Lastly, only *Bmal1* KO females in 21% O_2_ had increased epithelial thickness compared to WT females in 21% O_2_. Otherwise, differences in airway epithelial thickness did not yield significant effects, which is in line with previous findings (Bartman et al., 2024) (Figure 5c). Representative H&E images are shown (Figure 5d).

### 3.5 Extracellular matrix

**FIGURE 6:**
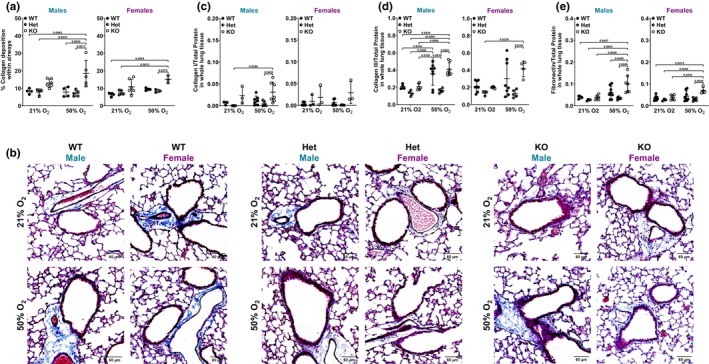
Extracellular matrix. (a) MT staining of FFPE sections were used to assess collagen deposition within airways. Quantification was done using Orbit Image Analysis software using a pixel‐based classification model to differentiate amount of collagen versus background within specific airways (as previously described (Bartman et al., 2024)). The resulting ratio was multiplied by 100. (b) Representative images are shown: collagen (blue) and noncollagen (red/pink). ImageJ was used to adjust white balance for consistency. Data are represented as mean ± SD; *n* = 3–6 pups per group. Protein expression of (c) collagen I, (d) collagen III, and (e) fibronectin from whole lung tissue of male and female *Bmal1* WT, Het, and KO neonates was measured using an automated and quantitative immuno‐capillary assay (Jess by ProteinSimple). Molecular weight used for peak detection was ~150 kDa for collagen I, ~280 kDa for collagen III, and ~280 kDa for fibronectin. Protein expression was normalized to total protein. Two‐way *ANOVA* with Tukey's multiple comparison test was used to compare protein expression in whole lung tissue between *Bmal1* genotypes and O_2_ exposure groups based on sex. Data are represented as mean ± SD; *n* = 3–8 pups per group.

We previously showed that 50% O_2_ alters airway extracellular matrix using *in vitro* (human ASM cells) (Britt Jr. et al., 2015; Vogel et al., 2017) and in vivo (neonatal hyperoxia) (Bartman et al., 2024; Faksh et al., 2016) models. Additionally, we recently showed by MT staining that female neonates have increased collagen deposition following 50% O_2_ exposure compared to females in 21% O_2_ (Bartman et al., 2024). Here, MT staining showed increased collagen deposition in *Bmal1* WT female neonates exposed to 50% O_2_ (albeit not significant), an effect that was not demonstrated by WT males exposed to 50% O_2_ (Figure 6a). *Bmal1* KO male neonates in 50% O_2_ showed increased airway collagen deposition compared to both WT and Het male neonates in 21% and 50% O_2_. *Bmal1* KO males in 21% O_2_ had increased collagen deposition, but it was not significant. *Bmal1* KO female neonates exposed to 50% O_2_ had increased collagen deposition compared to Het females in 50% O_2_ and compared to WT and Het females in 21% O_2_ (Figure 6a). Representative images are shown (Figure 6b).

Protein expression of ECM markers, collagen I, collagen III, and fibronectin in whole lung tissue were also evaluated. Female neonates did not show large differences in collagen I protein expression, regardless of genotype. *Bmal1* KO male neonates exposed to 50% O_2_ had increased collagen I protein expression compared to Het males in 21% or 50% O_2_ (Figure 6c). *Bmal1* WT male neonates showed increased collagen III protein expression in 50% O_2_ compared to Het males in 50% O_2_ and compared to WT, Het, and KO males in 21% O_2_. *Bmal1* Het male neonates exposed to 50% O_2_ had lower collagen III protein expression compared to WT males in 50% O_2_. *Bmal1* WT males had increased collagen III in 50% O_2_ compared to WT, Het, and KO males in 21% O_2_. *Bmal1* KO female neonates had increased Collagen III protein expression compared to Het females in 21% or 50% O_2_ (Figure 6d). Lastly, *Bmal1* KO male neonates exposed to 50% O_2_ had increased fibronectin protein expression compared to WT and Het males in 50% O_2_ and compared to WT, Het, and KO males in 21% O_2_. *Bmal1* KO female neonates had increased fibronectin protein expression compared to Het females in 50% O_2_ and compared to WT, Het, and KO females in 21% O_2_ (Figure 6e).

## 4 DISCUSSION

Understanding the mechanisms of hyperoxia insults during the neonatal period in the premature developing airway that contribute to chronic airway disease is critical for improving therapeutic outcomes for infants and children. To our knowledge, this is the first study to merge our neonatal moderate hyperoxia model with *Bmal1* KO mice to assess lung structure and function prior to adulthood. Our study highlights important factors to consider in understanding effects of moderate O_2_ on neonatal lung structure and function: sex and *Bmal1*. This study is intended to lay the groundwork for future studies to incorporate clock biology into our understanding of the premature developing lung.

We show that *Bmal1* WT females, but not males, are sensitive to O_2_ exposure: WT females exposed to 50% O_2_ from P0 to P7 have increased Rrs and decreased Crs at P21 compared to WT females exposed to 21% O_2_ from P0 to P21 (Figure 3a,b). Interestingly, *Bmal1* Het males and females did not follow the same pattern as the WT neonates: neither male nor female Het neonates showed sensitivity to O_2_ in terms of Rrs (Figure 3b), and Het males exposed to 50% O_2_ had slightly elevated Crs (Figure 4b). *Bmal1* KO male neonates exposed to 50% O_2_ from P0 to P7 showed increased Rrs (Figure 3c) and decreased Crs (Figure 4c) at P21 compared to KO males exposed to 21% O_2_ from P0 to P21. On the contrary, KO females exposed to 50% O_2_ had lower Rrs compared to KO females in 21% O_2_ (Figure 3c) without a difference in Crs (Figure 4c).

Functional studies were supported by structural and molecular analyses (airway, ASM thickness; collagen deposition). Notably, airway and ASM thickness in male and female *Bmal1* genotypes in both O_2_ exposure groups align with Rrs response to MCh challenge. Airways are thicker in *Bmal1* KO male neonates exposed to 50% O_2_ compared to KO males in 21% O_2_ and compared to Het males in 21% or 50% O_2_ (Figure 5a). ASM is thicker in KO males exposed to 50% O_2_ compared to all other groups (Figure 5b). In line with these findings, KO males exposed to 50% O_2_ have increased Rrs compared to KO males exposed to 21% O_2_, and compared to male WT or Het neonates exposed to 50% O_2_ (Figure 3). Interestingly, airways are thicker in *Bmal1* KO females in 21% O_2_ (not 50% O_2_ as in the male KOs), compared to KO females in 50% O_2_ and compared to WT and Het females in 21% O_2_ (Figure 5a). ASM is thicker in KO females in 21% O_2_ compared to WT and Het females in 21% O_2_ (Figure 5b). These findings are consistent with increased Rrs in *Bmal1* KO females in 21% O_2_ (Figure 3). Lastly, WT males do not show sensitivity to 50% O_2_ exposure in terms of Rrs, whereas WT females have increased Rrs in 50% O_2_ (Figure 3). This is supported by our data showing that WT males did not have measurably thicker airways or ASM in 50% O_2_, but the WT females did in fact have thicker airways (Figure 5a).

Our data follow a general pattern: *Bmal1* WT females are sensitive to 50% O_2_ with loss of that sensitivity in KO females, whereas males do not show O_2_ sensitivity in *Bmal1* WT but do show O_2_ sensitivity in *Bmal1* KO. This “reversal” (i.e., opposite protective/deleterious impact of *Bmal1* genotype) is not necessarily a new phenomenon. In one study using non‐tumorigenic human mammary epithelial cells (MCF10A) and invasive tumorigenic mammary epithelial‐like cells (MDA‐MB‐231), *Bmal1* knockout had opposing effects: on one hand, *Bmal1* KO sensitized both cell lines to genotoxic agents thus driving apoptosis and reflecting an anticancer effect. On the other hand, *Bmal1* KO increased MDA‐MB‐231 cells invasion potential thus exhibiting a pro‐cancer effect (Korkmaz et al., 2018). Additionally, loss of renal *Bmal1* has a differential effect on blood pressure based on sex, where *Bmal1* KO lowers blood pressure in male, but not female, mice. This is likely attributed to suppressed epithelial Na+ channel (ENaC) activity in only male *Bmal1* KO mice (Crislip et al., 2020). A study where *Bmal1* was deleted in the intestine showed an effect only once mice were put on a high‐fat diet under which the intestinal *Bmal1* KO was protective against obesity, hyperlipidemia, and fatty livers (Yu et al., 2021). This is in contrast to *Bmal1* KO in hepatocytes promoting hyperlipidemia (Pan et al., 2016), and *Bmal1* KO in adipocytes driving obesity (Paschos et al., 2012). Furthermore, while progression of liver fibrosis in humans is associated with a decrease in expression of BMAL1‐regulated genes, *Bmal1* KO mice are protected against liver fibrosis, inflammation, and steatohepatitis, even though these mice are prone to obesity (Jouffe et al., 2022). Also, these findings suggest sex hormones are involved in the protective effects of *Bmal1* KO in the liver (Jouffe et al., 2022). Taken together, these studies highlight the context‐ and tissue‐specific nature of BMAL1.

Because the goal of this study was to first establish the relevance of BMAL1 in our neonatal moderate hyperoxia exposure model, we investigated the independent role of BMAL1 in neonatal hyperoxic lung injury by performing all our studies in a morning time range (8 am–12 pm). However, there are limitations to this approach. Because BMAL1 is inherently part of cellular clock networks, and this component is necessary for maintaining rhythmicity in cellular and physiological systems (Albrecht, 2004; Bartman et al., 2020; Dibner et al., 2010), our study does not include assessment of circadian rhythms in neonatal mice, or the effect of O_2_ on neonatal circadian rhythmicity. For circadian rhythm studies, it is particularly important to have precise time‐point measurements throughout at least 1 day. While that approach will address circadian rhythmicity in the neonate, it presents logistical challenge when it comes to measuring lung mechanics (e.g., the time it takes during MCh challenge on the flexiVent). Harvesting neonatal mice at specific time intervals also poses a challenge due to the number of litters needed to obtain sufficient genotypes for each sex exposed to both O_2_ conditions at each time point. However, when and how circadian rhythms in the neonatal lung are established during the perinatal period, and whether synchrony versus asynchrony is important during fetal or neonatal development, are worthy questions to investigate. Adult *Bmal1* KO mice lack circadian rhythmicity in behavior (e.g., wheel running activity, sleeping, and eating) (Bunger et al., 2000), but this has not been studied in neonates. Interestingly, there is altered *Bmal1* gene expression rhythmicity in lung explants from adult mice exposed to high O_2_ (95%) at neonates (Issah et al., 2021), supporting the need to understand circadian rhythms in the neonatal lung following O_2_ exposure. Additionally, gene dosage is an important factor to consider in terms of how circadian rhythmicity would be affected (i.e., *Bmal1* Het neonates may have altered rhythms compared to both WT and KO, thus affecting interpretations of O_2_ effects). It is thought that clock genes are expressed early on in embryonic development, and that maternal factors influence fetal clocks, but there is limited information on the timing of clock rhythms in the multitude of lung cell types and their functional role in perinatal lung development (Astiz & Oster, 2020; Seron‐Ferre et al., 2007). The present study aimed to establish relevance of *Bmal1* in the developing airway exposed to moderate O_2_, and suggest future investigation into circadian rhythm in the lungs of neonates exposed to hyperoxia is warranted.

Once we understand mechanisms of clock biology in the developing lung, we can then begin to leverage these endogenous networks to reverse or attenuate effects of O_2_ on the premature airway. One way to target BMAL1 and its downstream clock networks is through clock modulators or small molecular ligands. For example, the nuclear receptor REV‐ERBα (*NR1D1*) has a variety of readily available small molecule compounds that are of interest for targeting the clock. This is because REV‐ERBα is involved in cellular clock regulation: REV‐ERBα inhibits *Bmal1* gene expression via ROREs, and BMAL1 transcription factor activity regulates expression of *NR1D1*. Thus, modulation of REV‐ERBα (via agonists or antagonists such as GSK4112, SR9009, GSK9245, and SR8278 (Wang et al., 2020)) influences BMAL1 and clock networks. Additionally, dexamethasone (Bartman et al., 2021) and forskolin are commonly used in lab settings to manipulate clock oscillations, and thus would be excellent starting points for experimental studies in terms of developing lung and moderate O_2_ exposure. Another unique way to target clocks is through the use of temperature or O_2_ itself. A recent study showed that temperature cycling was sufficient to synchronize core clock genes in human pediatric airway epithelial cells differentiated at an air‐liquid interface (Powell et al., 2022). Additionally, clocks and oxygen‐sensing pathways are evolutionarily linked (Bartman et al., 2020; O'Connell et al., 2020). From a mechanistic standpoint, core clock genes are altered expression following O_2_ exposure while cycling O_2_ levels is sufficient to synchronize cellular clocks in a manner dependent on hypoxia inducible factor 1α (HIF1α) (Adamovich et al., 2017, 2019; Peek et al., 2017). In fact, studies have shown that HIF1α transcriptionally controls expression of core clock genes and is therefore involved in oscillatory patterns (Adamovich et al., 2017; Manella et al., 2020; Peek et al., 2017). In our recent *in vitro* study using human fetal ASM, we showed clock gene expression sensitivity to O_2_ such that O_2_ may indeed be altering the clock (Bartman et al., 2021). Clinically, when neonates are administered supplemental O_2_, they experience shifts in O_2_ saturation leading to intermittent hypoxia‐hyperoxia (Zhang et al., 2023). It is therefore important to consider not only the percentage of oxygen in experimental studies, but also the timing (e.g., chronic versus intermittent) and cyclicity of it as well. If O_2_ is sufficient to target the clock, perhaps modulating O_2_ exposure in the NICU can offer a therapeutic advantage. Mechanistic studies are necessary to gain a deeper understanding of the link between O_2_ and the lung clock.

Clinically, it's also important to consider sex differences in neonatal lung injury and chronic airway disease: male preterm infants have poorer outcomes compared to females (Halvorsen et al., 2005). Our findings indicate that neonatal sex is an important factor in understanding O_2_ effects in the developing lung. We show that moderate hyperoxia deleteriously affects *Bmal1* WT female neonatal lung function (WT males did not show O_2_ sensitivity), and that males were deleteriously impacted by O_2_ in the *Bmal1* KO group (KO females did not show the same susceptibility to O_2_), findings of which are supported by histological analyses of airway thickness, ASM thickness, and collagen deposition. During the neonatal period, sex differences most likely result from sex chromosomes rather than sex hormones, but maternal sex hormones could play a role prenatally. Recent mouse studies identified sex‐specific differences in neonatal hyperoxic‐induced lung injury, with a key role of chromosomal versus hormonal sex (Cantu, Gutierrez, et al., 2023; Grimm et al., 2021). This study used a BPD neonatal hyperoxia model, which uses high O_2_ (95%) rather than moderate O_2_, and combined it with the Four Core Genotypes mice (four mouse strains with female or male chromosomal sex, each with either female or male sex hormones). Results from these studies showed that the male sex, regardless of sex hormones present, deleteriously impacts hyperoxia effects on factors relevant to BPD (alveolarization and vascularization) in the neonatal lung (Grimm et al., 2021). Furthermore, this was supported by single‐cell resolution of all lung cell subpopulations, which showed differences in transcriptional changes and endothelial to mesenchymal transition following high O_2_ exposure based on neonatal sex (Cantu, Cantu Gutierrez, et al., 2023; Cantu, Gutierrez, et al., 2023). Our studies were not BPD‐focused and therefore did not use high O_2_ or assess alveolarization or vascularization. However, these findings highlight that understanding chromosomal sex differences compounded by perinatal insults such as hyperoxia (and potentially involvement of cellular clocks) is worthy of further inquiry.

In summary, we initially proposed that clock component BMAL1 would be important in the developing lung. This study provides characterization of the *Bmal1* KO mouse model exposed to moderate hyperoxia during the neonatal period. Our use of functional, structural, and molecular approaches revealed differential effects of *Bmal1* and O_2_ based on sex. This work serves as a foundation for deeper exploration into clocks in the premature developing lung and in the context of moderate O_2_. These findings highlight the importance of considering neonatal sex and *Bmal1* (and potentially clock biology) in effort to understand neonatal hyperoxic lung injury toward future therapeutic strategies for the premature developing airway.

## AUTHOR CONTRIBUTIONS

C.M.B., C.P., and Y.S.P. conceived and designed research; C.M.B., L.N., K.K.L., L.K., and Y.F. performed experiments; C.M.B. analyzed data; C.M.B., C.P., and Y.S.P. interpreted results of experiments; C.M.B. prepared figures; C.M.B. drafted manuscript; C.M.B., L.N., K.K.L., L.K., Y.F., C.P., and Y.S.P. edited and revised manuscript; C.M.B., L.N., K.K.L., L.K., Y.F., C.P., and Y.S.P. approved final version of manuscript.

## FUNDING INFORMATION

These studies are supported by American Heart Association Grant 20POST35210002 (C. M. Bartman), T32 HL105355 (C. M. Bartman), the Mayo Clinic Specialized Center of Research Excellence (SCORE) and Women's Health Research Center Career Enhancement Core Award U54 AG044170 (C.M. Bartman), and NIH National Heart, Lung, and Blood Institute Grants R01 HL160570 (C. Pabelick), and R01 HL056470 (Y. S. Prakash).

## CONFLICT OF INTEREST STATEMENT

No conflicts of interest, financial, or otherwise are declared by the authors.

## ETHICS STATEMENT

The animal study protocol was approved by the Institutional Animal Care and Use Committee (IACUC) at Mayo Clinic and performed in accordance with the National Institutes of Health's Guide for the Care and Use of Laboratory Animals.

## Supporting information


Figure S1

## Data Availability

Data will be made available upon reasonable request. Figure S1 is provided via Figshare (https://doi.org/10.6084/m9.figshare.c.7249897).
