# Supplementary material for: BMAL1 sex‐specific effects in the neonatal mouse airway exposed to moderate hyperoxia
Source: Physiol Rep. 2024 Jun 28;12(13):e16122. doi: 10.14814/phy2.16122 (PMC11213646; doi:10.14814/phy2.16122)
Supplement: Supplementary file 1 — Figure S1 [file PHY2-12-e16122-s001.zip › phy216122-sup-0002-FigureS1.pdf]

Supplemental Figure

Figure S1. (A) Elastance, (B) Tissue Damping, (C) Elasticity, (D) Inspiratory capacity from lung mechanics (flexiVent) analysis of P21 male and female neonates during MCh challenge of **(i) *Bmal1* WT neonates, (ii) *Bmal1* Het neonates, and (iii) *Bmal1* KO neonates.** Effect of 21% vs 50% O_2 in each sex is shown pairwise on graphs. **+** = Effect of female vs male in 21% O_2 (same genotype in each comparison; represented in blue on female graph); **#** = Effect of female vs male in 50% O_2 (same genotype in each comparison; represented in blue on female graph). **%** = Effect of Het vs WT in 21% O_2 (same sex in each comparison); **&** = Effect of Het vs WT in 50% O_2 (same sex in each comparison). **†** = Effect of KO vs WT in 21% O_2 (same sex in each comparison); **‡** = Effect of KO vs WT in 50% O_2 (same sex in each comparison). **$** = Effect of KO vs Het in 21% O_2 (same sex in each comparison); **!** = Effect of KO vs Het in 50% O_2 (same sex in each comparison). **(i-iii)** Two-way *ANOVA* was used to compare MCh dose response curve of each *Bmal1* genotype and O_2 exposure group based on sex. **(iv-v)** Two-way *ANOVA* with Tukey's multiple comparison test was used to compare lung mechanics at baseline and maximum responses to MCh among *Bmal1* genotypes and O_2 exposure groups based on sex. **(i-v)** Data are represented as mean ± SD; *n* = 3-12 pups per group.
