# Supplementary figures and images for: BMAL1 sex‐specific effects in the neonatal mouse airway exposed to moderate hyperoxia
Source: Physiol Rep. 2024 Jun 28;12(13):e16122. doi: 10.14814/phy2.16122 (PMC11213646; doi:10.14814/phy2.16122)

A

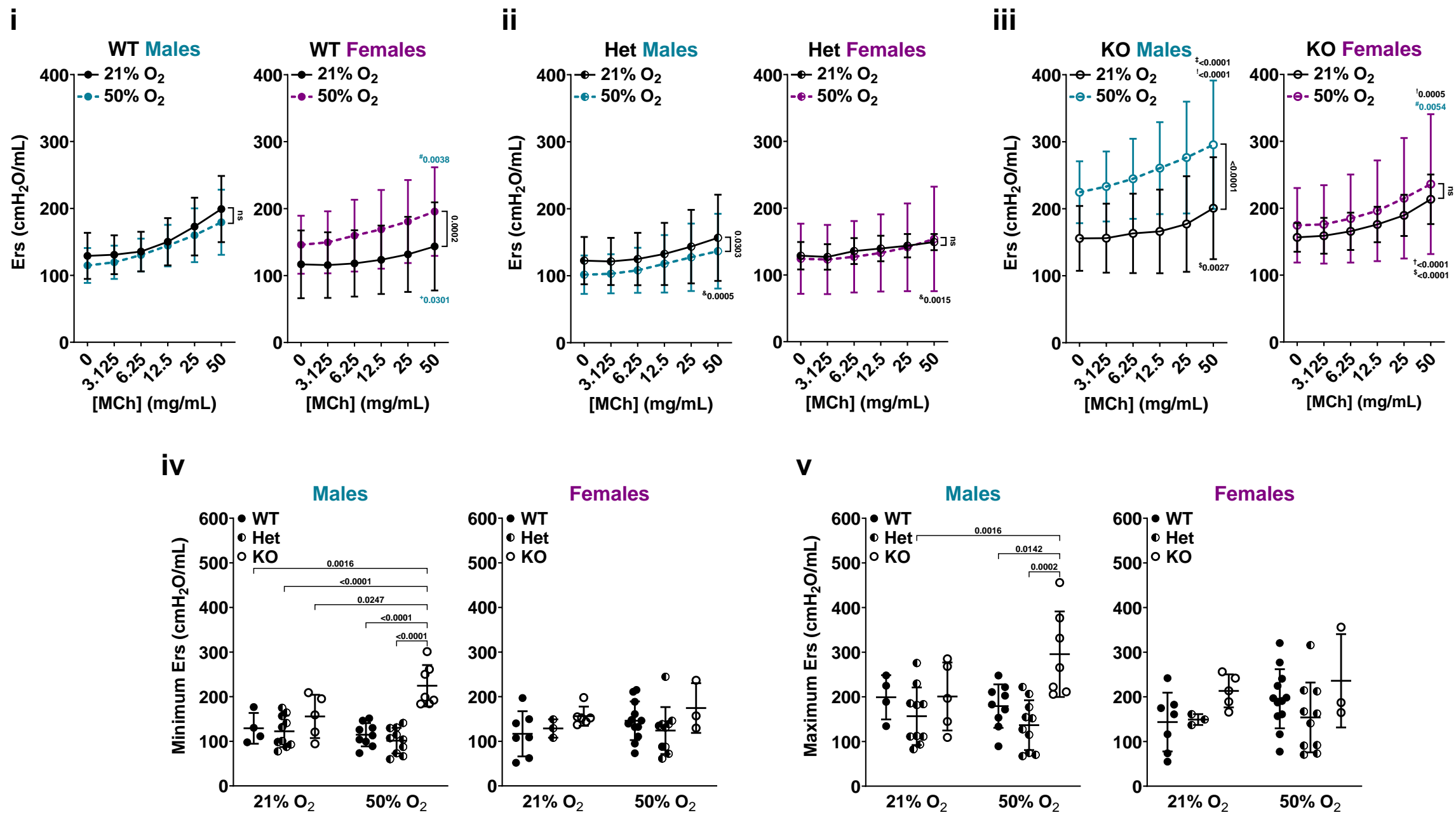

B

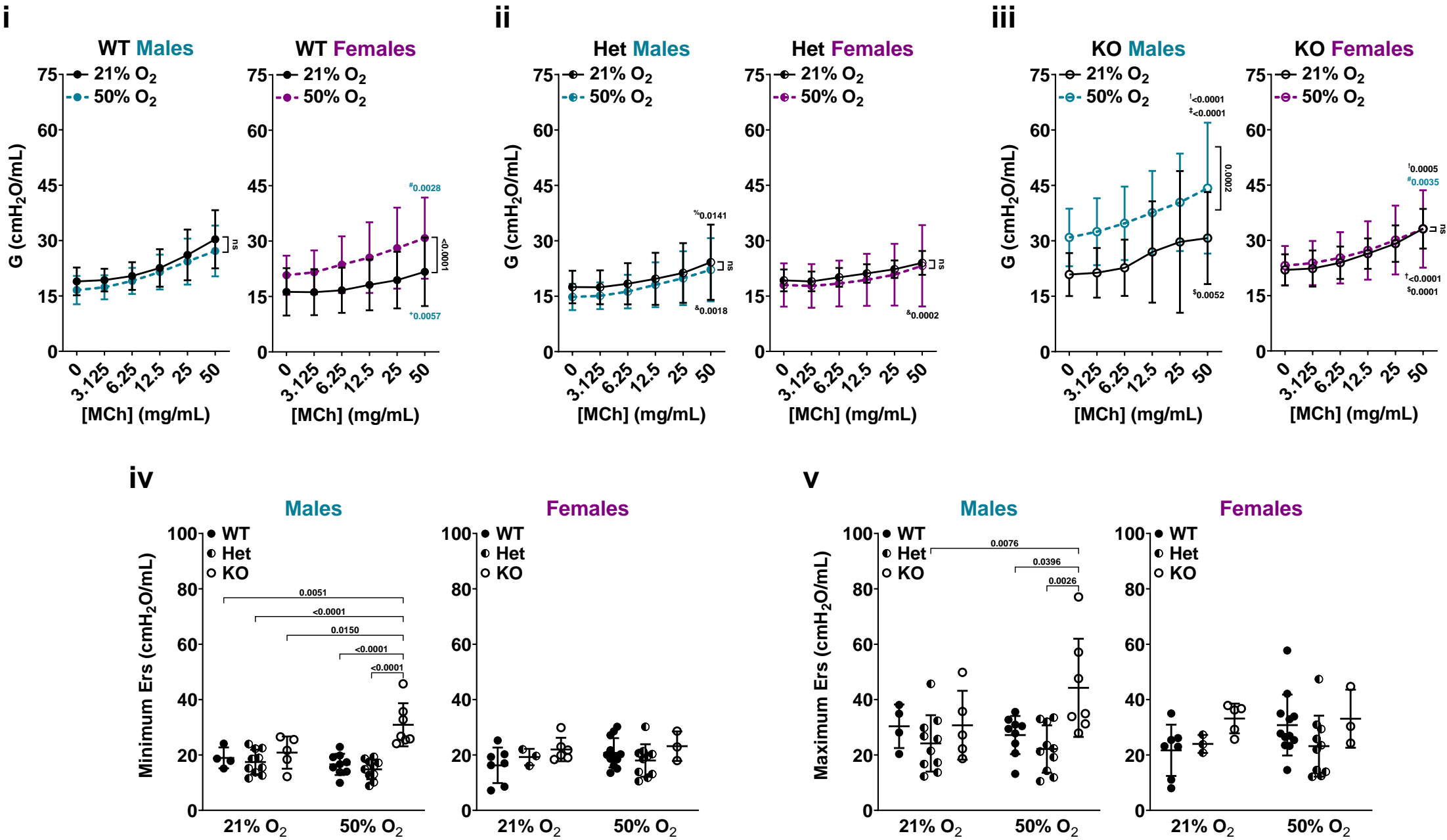

C

i

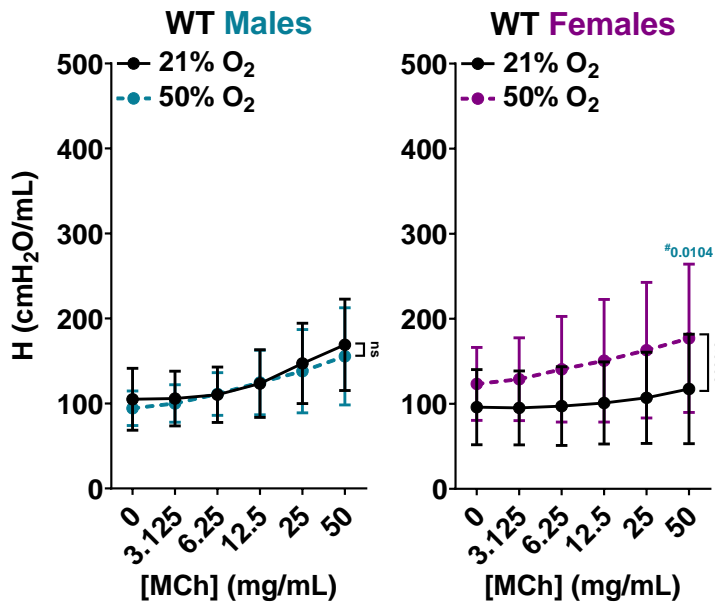

ii

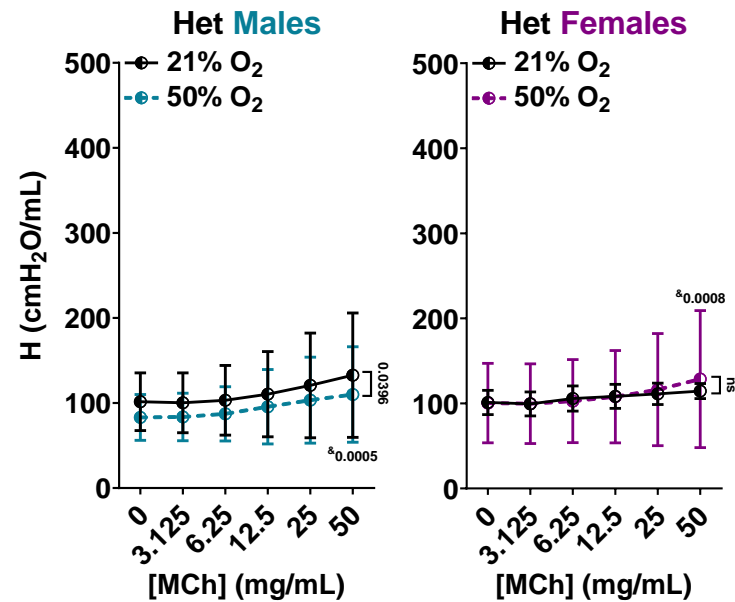

iii

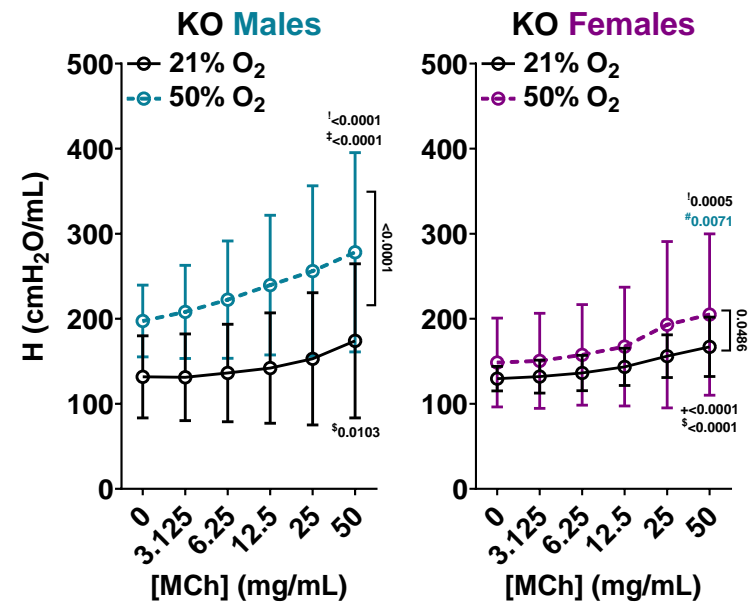

iv

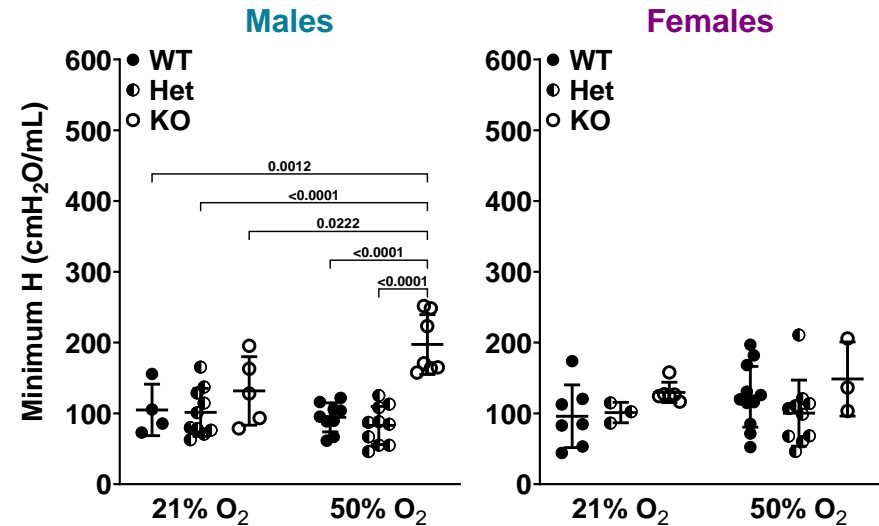

v

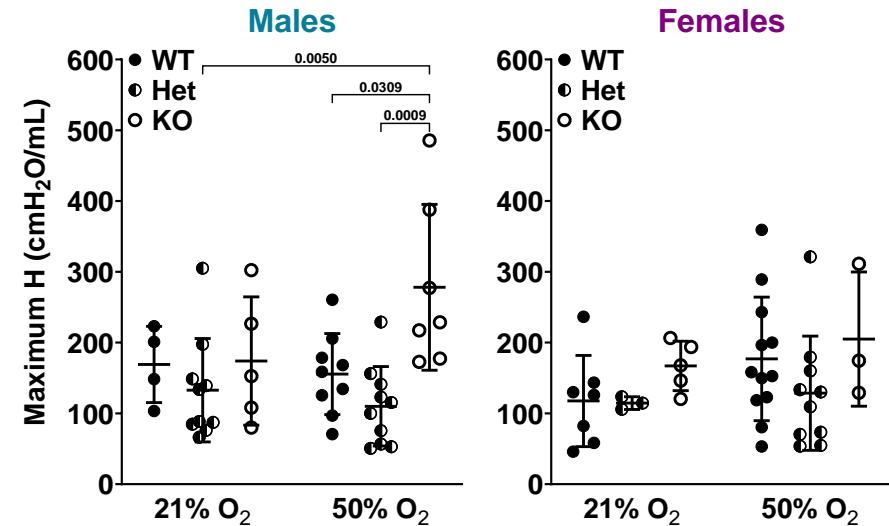

D

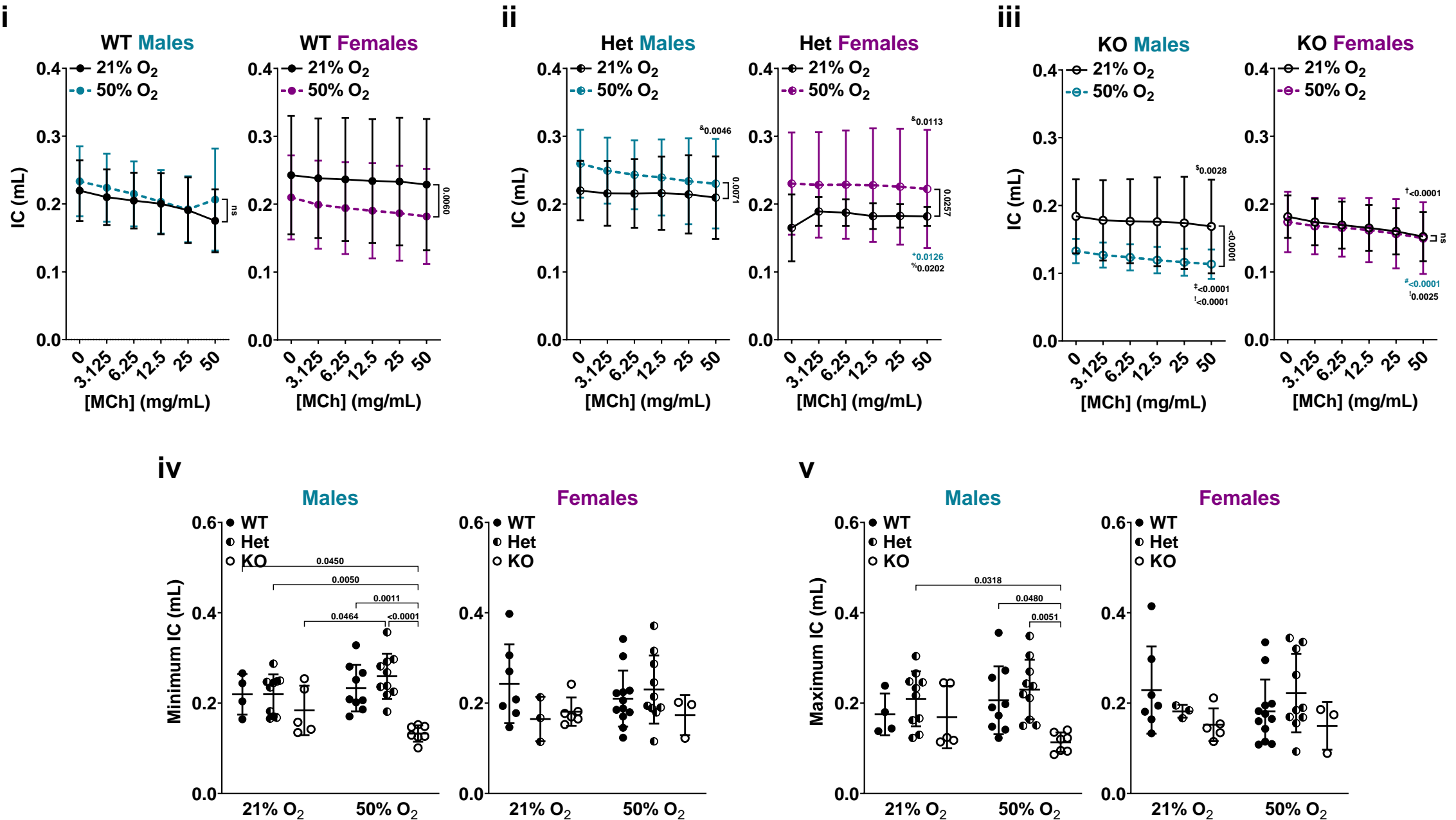

Supplement: Supplementary file 1 — Figure S1 [file PHY2-12-e16122-s001.zip › phy216122-sup-0001-Supinfo01.pdf]
